# Spatial organization of the budding yeast genome in the cell nucleus and identification of specific chromatin interactions from multi-chromosome constrained chromatin model

**DOI:** 10.1371/journal.pcbi.1005658

**Published:** 2017-07-13

**Authors:** Gamze Gürsoy, Yun Xu, Jie Liang

**Affiliations:** The Richard and Loan Hill Department of Bioengineering, Program in Bioinformatics, University of Illinois at Chicago, Chicago, Illinois, United States of America; Iowa State University, UNITED STATES

## Abstract

Nuclear landmarks and biochemical factors play important roles in the organization of the yeast genome. The interaction pattern of budding yeast as measured from genome-wide 3C studies are largely recapitulated by model polymer genomes subject to landmark constraints. However, the origin of inter-chromosomal interactions, specific roles of individual landmarks, and the roles of biochemical factors in yeast genome organization remain unclear. Here we describe a multi-chromosome constrained self-avoiding chromatin model (mC-SAC) to gain understanding of the budding yeast genome organization. With significantly improved sampling of genome structures, both intra- and inter-chromosomal interaction patterns from genome-wide 3C studies are accurately captured in our model at higher resolution than previous studies. We show that nuclear confinement is a key determinant of the intra-chromosomal interactions, and centromere tethering is responsible for the inter-chromosomal interactions. In addition, important genomic elements such as fragile sites and tRNA genes are found to be clustered spatially, largely due to centromere tethering. We uncovered previously unknown interactions that were not captured by genome-wide 3C studies, which are found to be enriched with tRNA genes, RNAPIII and TFIIS binding. Moreover, we identified specific high-frequency genome-wide 3C interactions that are unaccounted for by polymer effects under landmark constraints. These interactions are enriched with important genes and likely play biological roles.

## Author summary

The architecture of the cell nucleus and the spatial organization of the genome are important in determining nuclear functions. Single-cell imaging techniques and chromosome conformation capture (3C) based methods have provided a wealth of information on the spatial organization of chromosomes. Here we describe a multi-chromosome ensemble model of chromatin chains for understanding the folding principles of budding yeast genome. By overcoming severe challenges in sampling self-avoiding chromatin chains in nuclear confinement, we succeed in generating a large number of model genomes of budding yeast. Our model predicts chromatin interactions that have good correlation with experimental measurements. Our results showed that the spatial confinement of cell nucleus and excluded-volume effect are key determinants of the folding behavior of yeast chromosomes, and largely account for the observed intra-chromosomal interactions. Furthermore, we determined the specific roles of individual nuclear landmarks and biochemical factors, and our analysis showed that centromere tethering largely determines inter-chromosomal interactions. In addition, we were able to infer biological properties from the organization of modeled genomes. We found that the spatial locations of important elements such as fragile sites and tRNA genes are largely determined by the tethering of centromeres to the Spindle Pole Body. We further showed that many of these spatial locations can be predicted by using the genomic distances to the centromeres. Overall, our results revealed important insight into the organizational principles of the budding yeast genome and predicted a number of important biological findings that are fully experimentally testable.

## Introduction

Understanding the spatial organization of the genome in the cell nucleus is essential to gain insight into important nuclear activities such as repair, recombination, and replication of DNA, as well as the control of the transcriptional status of genes [1, 2]. The overall organization of genome has been shown to be compartmentalized in the form of chromosome territories [3], topologically associated domains [4, 5], and spatial localization of individual gene loci [6]. Such compartmentalization affects the expression levels of genes among eukaryotes from yeast [2] to mammals [1]. With its well understood nuclear architecture and transcriptional machineries [2], budding yeast provides an excellent model system for investigating how eukaryotic cellular activities are related to genome organization. Furthermore, there is now clear evidence that important nuclear events such as cancer-promoting chromosomal translocations observed in human nuclei and relocation of genomic elements upon breaks of double stranded DNA observed in budding yeast originate from analogous cellular machineries [2].

Studies using electron microscopy techniques revealed detailed structures of architectural landmarks of budding yeast nucleus. These include the spindle pole body (SPB), the nucleolus, and the nuclear envelope (NE) [6–14]. SPB is functionally equivalent to centrosome in mammalian nuclei, where all heterochromatic centromeres are attached throughout interphase [11]. The nucleolus, where ribosome synthesis and assembly take place, contains clusters of ribosomal DNA (rDNA) repeats [6, 10, 12–15]. The NE, where telomeric regions of yeast chromosomes are anchored, facilitates silencing of telomeric genes [7–10]. In addition, microscopy experiments further revealed the dynamics behavior of important genes of budding yeast [6].

The spatial organization of the yeast genome likely results from both generic polymer effects such as self-avoiding polymer chains confined in the cell nucleus [2, 25, 27, 32, 33] as well as effects of biochemical factors such as transcription factor binding [2]. With genome-wide studies using Chromosome Conformation Capture (3C) technique [16, 17], large-scale long-range chromatin looping interactions across the budding yeast genome have been identified [16]. Studies of polymer models of both human [18–25] and yeast [26–30] genomes have revealed important insight into the principle of genome folding. For example, two recent seminal computational studies demonstrated that chromosomes of budding yeast behave as randomly folded flexible self-avoiding polymer chains that are subject to the constraints of nuclear landmarks and nuclear confinement [26, 27]. It was shown that tethering of genomic elements such as centromeres and telomeres to the nuclear landmarks gives rise to the preferential localization of chromosomes and functional loci in the nucleus [26, 27]. In addition, modeled interactions are found to have excellent correlation with experimentally captured interactions at the chromosome level, with intra-chromosomal locus-locus interactions well reproduced at 32–75 kb resolution [26, 27]. However, modeled and experimentally captured inter-chromosomal interactions are only modestly correlated. Furthermore, these volume exclusion models [26, 27] may be capturing only interactions arising from generic polymer effects, with strong correlation (R>0.90) found only at lower resolution that requires binning of the interaction frequency data. A recent study showed that after correction of measured interaction frequencies using a statistical null model, the budding yeast genome no longer exhibits properties of a randomly folded polymer under constraints [31]. The important issue whether the organization of the yeast genome is dictated by physical tethering of landmarks and the excluded-volume effects as discussed in [26, 27], with specific protein-mediated interactions playing negligible roles, remains uncertain. Overall, the precise roles of nuclear landmarks, volume confinement, biochemically mediated interactions, as well as their relative contributions to the overall organization of yeast genome are unclear.

In this study, we explored computationally the structural properties of budding yeast genome under different combinations of landmark constraints and nuclear confinement. Our goal is to answer the following questions: (1) how does the confinement of the cell nucleus affect the organization of the yeast genome, (2) to what extent the genome organization determined by the physical architecture of the nucleus, (3) what are the relative contributions of the individual nuclear landmarks to overall genome organization, (4) how can we distinguish chromatin looping interactions arising from biochemical factors from those arising from generic polymer properties. Our study is based on the multi-chromosome Constrained Self-Avoiding Chromatin (mC-SAC) method and the generation of ensembles of ∼150,000 model genomes using the geometrical Sequential Importance Sampling technique (g-SIS) [32, 33].

In agreement with previous studies [26, 27], our results showed that indeed the overall patterns of chromatin interactions of the budding yeast genome are well captured when only polymer effects under the spatial confinement of cell nucleus and landmark constraints are considered, with now good correlation for both intra- and inter-chromosomal interactions at the improved resolution of 15 kb (row-based Pearson correlation coefficient *R* of 0.91). Our study further specified the roles of individual landmark constraints, and showed that the size of the nuclear confinement is the key determinant of intra-chromosomal interactions, while centromere tethering is responsible for much of the observed inter-chromosomal interactions and correlation of pairwise telomere distances to chromosomal arm lengths. Our study also shed some light on the origin of the spatial locations of eight important genes, as they can be determined by their genomic distances to the centromeres. In addition, we report a number of additional new findings. We found that chromosomal fragile sites where double-stranded DNA breaks upon DNA perturbation are clustered in three-dimensional space. Furthermore, novel chromatin interactions undetected in experimental studies [16] are uncovered from our computational ensemble models of yeast genomes. These novel chromatin interactions are enriched in tRNA genes and are found to be stabilized by binding of the transcription factors TFIIS and RNA polymerase III. Our results further indicate that the clustering of tRNA genes, to a large extent, is likely a consequence of the spatial clustering of centromeres to the SPB. In addition, we found there are interactions between specific genomic elements enriched with known important genes that were not captured by polymer properties, but are detected in experimental studies [16]. This was made possible by removing expected interactions due to polymer effects from experimental measurements. Overall, our findings define the specific roles of confinement and individual landmarks, and can uncover likely biologically relevant interactions from genome-wide 3C measurements that are beyond polymer effects.

## Materials and methods

### Model and parameters

**Fig 1:**
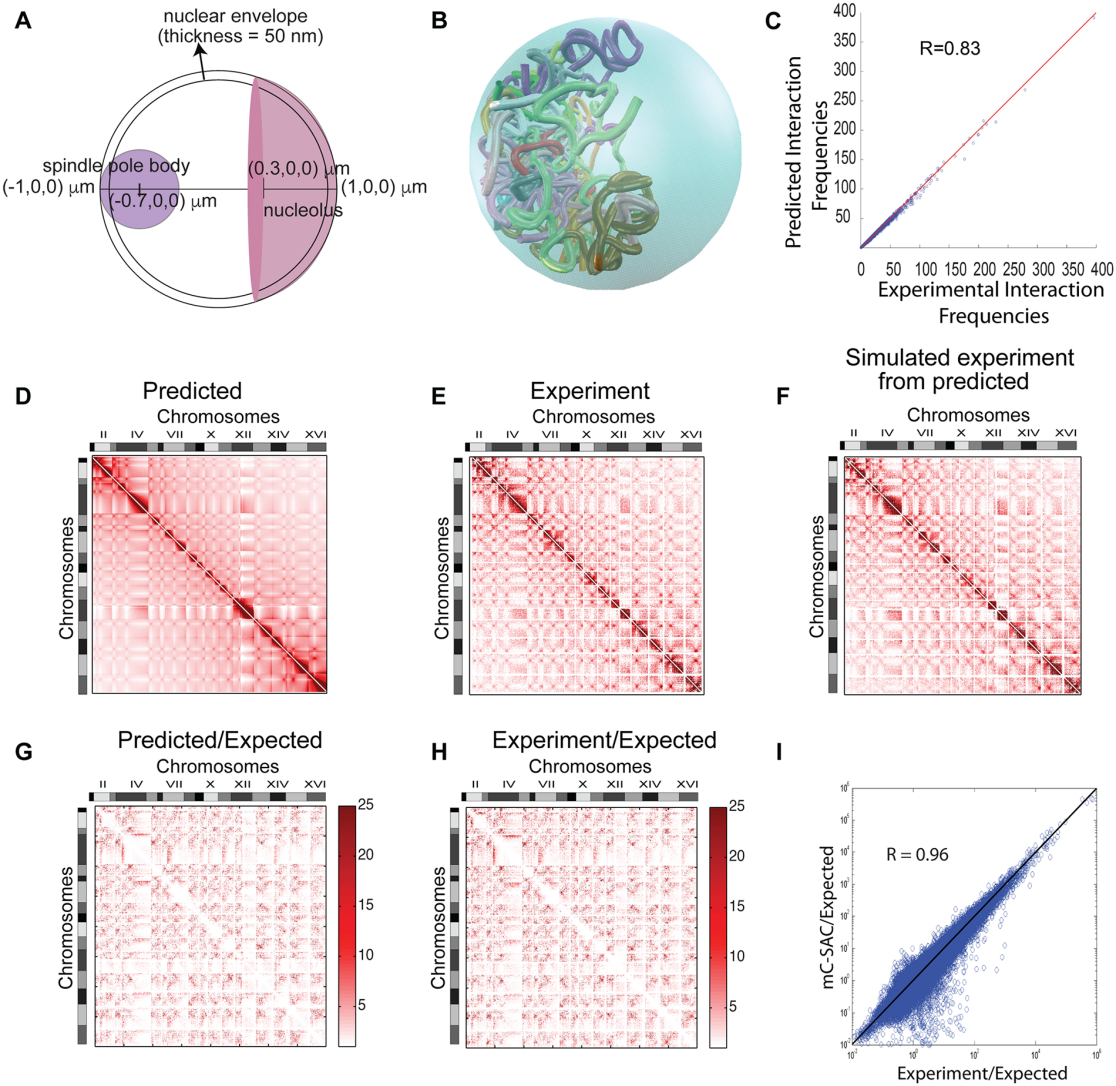
The nuclear architecture of budding yeast and the mC-SAC model genome. **(A)** Schematic representation of the nucleus and nuclear landmarks of budding yeast and their corresponding coordinates and dimensions (not to scale). **(B)** An example 3D structure of mC-SAC genome confined in the cell nucleus. **(C)** Correlation between genome-wide chromatin conformation capture interaction frequencies and interaction frequencies measured from the fully-constrained ensemble of model yeast genomes. **(D)** Heat map of interaction frequencies measured in the fully-constrained ensemble. Darker color indicates higher interaction frequency. **(E)** Heat map of interaction frequencies from the experimental measurements [16]. **(F)** Heat map of simulated interactions from the fully-constrained ensemble, with only interactions between restriction fragments of the genome-wide 3C experiment [16] are shown for direct comparison. **(G)** Heat map of interaction frequencies of the fully-constrained ensemble that are corrected after removal of expected interaction frequencies obtained from an ensemble generated using only nuclear confinement and excluded-volume as constraints. **(H)** Heat map of interaction frequencies of the genome-wide 3C experiments that are corrected after removal of expected interaction frequencies. **(I)** Correlation of interaction frequencies between genome-wide 3C data and from the fully-constrained ensemble, after removal of expected interactions as obtained from an ensemble generated using only nuclear confinement and excluded-volume as constraints.

The nuclear architecture of budding yeast is composed of Nuclear Envelope (NE), Spindle Pole Body (SPB), nucleolus, and 16 chromosomes. The locations of SPB, NE, and nucleolus are placed according to measurements from imaging studies (Fig 1A) [6–14]. The locations of the 16 chromosomes are modeled as independent but interacting polymers. Each monomer of the polymer chain is modeled as spheres that corresponds to a 3 kb of DNA [34, 36]. The entire budding yeast genome is modeled a total of 3990 monomers and are divided into 16 chromosomes.

### Chain growth by geometrical Sequential Importance Sampling (g-SIS)

The mC-SAC model is developed based on our single C-SAC chain growth model [32, 33]. First, we mapped the locations of centromeres, telomeres and rDNA repeats onto the polymer chains that corresponds to each chromosome. Each chromosome is divided into right and left arms from their centromeres, except Chr 12 (Fig A in S1 Text). The polymer chain representing Chr 12 is divided into three segments to accommodate the nucleolus (see S1 Text and Fig A in S1 Text).

The budding yeast genome is therefore composed of 33 chromosomal arms, each represented by a polymer chains. The genome *γ* = (*x*^1^, *x*^2^, …, *x*^33^) is a collection of chromosomal arms, where each arm *x*^*k*^ consists of *n* units as $x_{k}=\left(x_{1}^{k},x_{2}^{k},...,x_{n}^{k}\right)$. The three-dimensional location of the *i*-th unit of the *k*-th chromosome arm is denoted as $x_{i}^{k}=\left(a_{i}^{k},b_{i}^{k},c_{i}^{k}\right)\in R^{3}$.

To generate a chromosomal arm, we grow the mC-SAC chain one unit at a time (each unit contains 5 beads, i.e., 15 kb DNA), ensuring the self avoiding property along the way, namely, $x_{i}^{k}\neq x_{j}^{l}$ for all *i* ≠ *j*. We use a *s* = 1640-state off-lattice discrete model (see [32, 33, 37, 51] for more details). The new unit added to a partial chain is placed at $x_{t+1}^{k}$, taken from one of the unoccupied *s*-sites neighboring $x_{t}^{k}$, with a probability of growth *g*(***x***), which is the trial distribution. This selection introduce a bias away from the target distribution *π*(***x***), and this bias is corrected by assigning each successfully generated genome a proper weight *w*(***x***) = *π*(***x***)/*g*(***x***). Details can be found in references [32, 33, 37, 51].

The multiple chain growth process starts with a random selection of a chromosomal arm and placement of its corresponding centromere at a random location in the SPB. We then employ the chain growth strategy to grow chromosomal arms until the telomere of the corresponding arm reaches to the target location, *i*.*e*. NE. In the case of Chr 12, we select a random location on the nucleolus to place the rDNA repeats and grow the chain towards to its targeted location(*i.e.* NE or SPB). We repeat this process until all 33 chromosomal arms are completely generated (see S1 Text for details).

### Calculation of relative positions of genes

The relative positions of the genes with respect to the SPB is defined as the ratio between the median location of the gene and the median location of SPB in the ensemble of model mC-SAC genomes, namely,

$$\begin{array}{c} \text{median}(\text{gene})/\text{median}(\text{SPB})=\mu_{1/2}^{\text{gene}}/a_{\text{SPB}} \end{array}$$ (1)

where $\mu_{1/2}^{\text{gene}}$ is the median *a*_gene_ coordinate of the three-dimensional coordinates of **x**_gene_ = (*a*_gene_, *b*_gene_, *c*_gene_) calculated using the coordinates of ensemble of model genomes. The median coordinate of the SPB, *a*_SPB_, is pre-determined from the imaging experiments and depicted in Fig 1. This calculation is adopted from the original imaging study [6], where the three–dimensional coordinates were projected to two principal axis as (*ρ*_gene_, *z*_gene_), where *ρ*_gene_ corresponds to the projection of (*b*_gene_, *c*_gene_) and *z*_gene_ corresponds to *a*_gene_ (Fig A in S1 Text).

## Results

### mC-SAC model of budding yeast genome

We model the chromatin fiber of budding yeast as chained beads, where each bead corresponds to 3 kb of DNA with a diameter of 30 nm in accordance with the experimental and theoretical suggestions [34–36]. Following previous studies [26, 27, 33], we used light microscopy data to model the architecture of yeast nucleus. The nucleus is modeled as a sphere of a diameter of 2 *μ*m and contains the Spindle Pole Body (SPB), the Nuclear Envelope (NE, modeled as a shell of thickness of 50 nm following [26]), the nucleolus, and 16 chromosomes (Fig 1A and Fig A in S1 Text) [33]. Chromosomes all reside inside the nucleus as independent but interacting self-avoiding chromatin fibers. The entire budding yeast genome is represented by a total of 3,990 beads divided into 16 different chromosomes (Fig 1B).

An ensemble of ∼150,000 independent model genome structures are generated that are subject to the nuclear confinement, centromere clustering at SPB, telomere attachment at the NE, and rDNA repeat clustering at the nucleolus. This is achieved by sequentially growing self-avoiding chromatin chains one unit (5 beads) at a time, where each unit corresponds 15 kb of DNA using the technique of geometrical Sequential Importance Sampling (g-SIS) [32, 33, 37, 38]. We call this the *fully-constrained ensemble* of mC-SAC chains. In addition, we examined the effect of landmark constraints by generating separate ensembles of ∼150,000 independent model genomes. All of these ensembles are subject to nuclear confinement, excluded volume effect, and two or less constraints from nuclear landmarks (see Table 1). In total, we have 5 additional ensembles: (1) The ensemble of *without telomere* is subject to all landmark constraints except the telomere attachment to the NE, (2) the ensemble of *without nucleolus* is subject to all landmark constraints except the exclusion of chromatin in nucleolus, (3) the ensemble of *without centromere* is subject to all landmark constraints except the centromere tethering to the SPB, (4) the ensemble of *with only centromere* is subject to only centromere tethering to the SPB in addition to nuclear confinement and excluded volume effects, and (5) the *random* ensemble is only subject to nuclear confinement and excluded volume effects (see Table 1).

**Table 1:** The effects of different constraints on the folding of budding yeast genome: Row-based correlation coefficients between the interactions of model ensembles and the genome-wide chromosome conformation capture experiments at 15 kb resolution. Spatial confinement of a nucleus of diameter 2 *μ*m and excluded-volume effects are imposed in all cases.

	fully-constrained	without telomere	without nucleolus	without centromere	with only centromere	Random
Inter	**0.75**	0.74	0.72	0.30	0.86	0.25
Intra	**0.95**	0.95	0.95	0.87	0.93	0.89
Overall	0.91	0.91	0.91	0.90	0.77	0.77

### mC-SAC model with nuclear confinement and landmark constraints recapitulates long-range chromatin interactions of budding yeast genome

Recent genome-wide Chromosome Conformation Capture (3C) studies have quantified the frequency of chromatin looping interactions of budding yeast genome, which can be summarized by an interaction frequency matrix [16]. Two recent seminal studies showed that interactions at whole chromosome level, as well as intra-chromosomal locus-locus interactions at 32–75 kb resolution are well accounted for by polymer effects [26, 27].

To examine how well our model can capture the overall genome organization, we first calculated Pearson correlation between chromosome-pair interaction frequencies in the fully-constrained model ensemble and those detected in genome-wide 3C experiment [16]. The result of *R* = 0.99 at *p* < 7.08 × 10^−92^ is similar to those of previous studies [26, 27]. We then calculated the correlation between interaction frequency matrices following previous studies [26, 27]. The interaction frequency matrices obtained from our predicted ensemble (Fig 1D and 1F) and from genome-wide 3C experiments (Fig 1E) are strongly correlated, with an *R* = 0.83 at 15 kb resolution (Fig 1C, *p*-value ¡ 0.001, see also S1 Text and Fig B in S1 Text) following the calculation procedure of [27]. This is an improvement over *R*∼ 0.50 as reported in Figure S4 C of [27] at the same 15 kb resolution. The row-based R of 0.94 at 32 kb as calculated in [26] is also comparable with the reported R of 0.94 [26].

Importantly, the calculated inter-chromosomal interaction frequencies in the fully-constrained ensemble and those observed in genome-wide 3C experiments are also in agreement, with an R of 0.75 at 15 kb resolution. This compares favorably with previously reported R of 0.54 at a 2× lower resolution of 32 kb [26]. The heat maps obtained from experiments [16] and from mC-SAC ensemble have nearly identical patterns (Fig 1E–1F).

To eliminate the effect of proximity interactions and non-specific interactions arising from nuclear confinement of self-avoiding chromatin chains, we used our random ensemble as the null model to calculate the propensity (observed/expected) of each interaction in both fully-constrained ensemble (Fig 1H) and the genome-wide 3C data (Fig 1G). After exclusion of non-specific interactions, the propensities from the fully-constrained ensemble and propensities from genome-wide 3C measurements have strong correlation, with an R of 0.96 at 15 kb resolution and an R of 0.97 at 32 kb resolution (Fig 1I, see also S1 Text).

Overall, our results obtained from the fully-constrained models of budding yeast genome show that model genomes generated under the constraints of nuclear confinement and all three nuclear landmarks can capture much of the experimentally measured intra- and inter-chromosomal interactions at 15 kb resolution. Both experimentally measured and mC-SAC inter-chromosomal interactions are dominated by interactions between pericentromeric regions, hence a cross-like pattern originating from centromeres is observed (Fig 1D, 1E and 1F). These results suggest that nuclear confinement and nuclear landmarks play key roles in determining the overall organization of yeast genome.

### Nuclear size is a major determinant of overall spatial chromatin interactions in the budding yeast genome

#### Effects of confinement on patterns of genome-wide interactions

**Fig 2:**
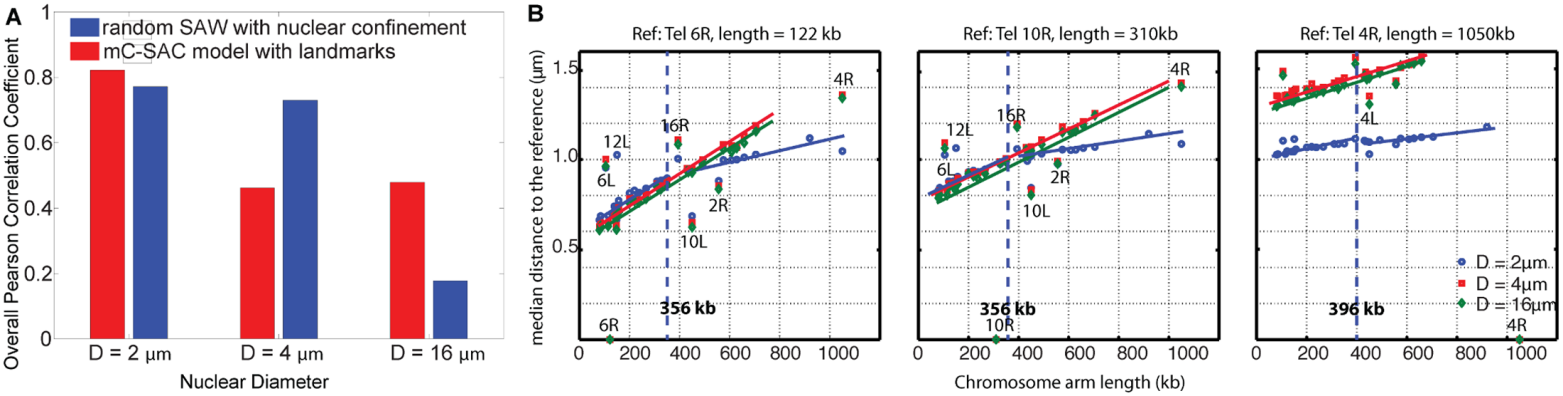
Effects of confinement on the overall folding behavior of budding yeast genome. **(A)** Overall correlation coefficient of the frequencies between genome-wide 3C measurements and modeled ensemble. As the nuclear size increases, correlation generally decreases. **(B)** Effects of nuclear size and chromosomal arm length on the median distances between telomeres. Relationships between arm length and median telomere distances at different nuclear sizes for the fully-constrained ensemble, with different telomeres as references are shown. Two linear regimens become one linear regime as *D* increases from 2 *μ*m to 4 and to 16 *μ*m.

To understand the effects of the nuclear confinement on chromatin interactions, we examined the frequency of interactions of model yeast genome with different degrees of confinement in nuclei of diameters of 2, 4 and 16 *μ*m, respectively, each with and without landmark constraints. A total of 6 ensembles, each with ∼150,000 model genomes are generated. As the nuclear diameter increases, the correlation between the interaction frequencies of fully-constrained ensemble and those of genome-wide 3C experiments decreases from R of 0.83 to 0.55 (Fig 2A). When the landmark constraints are removed, the interaction frequencies of random ensemble and frequencies of genome-wide 3C experiments decreases from R of 0.77 to 0.25 as the nuclear diameter increases from 2 *μ*m to 16*μ*m (Fig 2A). These results showed that the degree of confinement is a key determinant of the organization of budding yeast genome, as when only nuclear confinement constraint is employed, the correlation *R* is already quite strong at *R* = 0.77, so long as the appropriate confinement size is imposed.

#### Effects of confinement on pairwise distances between telomeres

Fluorescence imaging data suggested that telomeres are not randomly positioned on the nuclear periphery [39]. Instead, the spatial distance between any two telomeres increases gradually with the arm lengths of their chromosomes [39]. The fluorescence imaging data showed that distance between telomere pairs as a function of arm length can be divided into two linear regimes, with a change in the slope at around the arm length of between 266 and 394 kb for both right telomeres of Chr 6 and 4. However, no change point was detected for the right telomere of Chr 10. In addition, the short chromosome arm Tel 6R shows an increasing trend with respect to increasing arm length of other chromosomes, while the long chromosome arm Tel 4R shows the opposite trend [39]. Previous polymer studies also calculated the distance between telomere pairs as a function of arm length and succesfully captured the increasing trend with a change in slope in good agreement with the experiments [26]. Interestingly, they predicted increasing trend for every telomeres in budding yeast with a change in slope at 356 kb, when the genome is modeled as self-avoiding chromosomes subject to constraints of nuclear landmarks and confinement [26].

In this study, we further examined the origin of the correlation between the chromosomal arm lengths and the pairwise distances between telomeres by altering the nuclear size. Following ref. [26], we applied a change point analysis adopted from ref. [40] to the relationship between the median telomere-telomere distances and the chromosome arm lengths. In the fully-constrained ensemble at the nuclear diameter of 2 *μ*m, the median telomere-telomere distances and chromosome arm lengths are linearly correlated in two regimes, with a change in the slope at around 356 kb for Tel 6R and Tel 10R, and 396 kb for Tel 4R. (Fig 2B, blue dots and blue lines). This increasing trend of telomere-telomere distances with increasing length of chromosomal arms is in agreement with the experimental findings observed for Tel 6R and Tel 10R [39]. As in the case of previous constrained polymer models [26], our mC-SAC model found two linear regimes in the relationship of telomere-telomere distances with chromosomal arm lengths for Tel 10R. However, experimental findings suggested the existence of a single regime with no change in the slope. Similar to previous studies [26], our model also cannot reproduce the pattern of decreasing telomere-telomere distances with increasing arm lengths observed in Tel 4R. It was suggested that these disagreements could be due to the small number of samples in the experiments [26]. It is also possible that the discrepancy in the observed behavior of Tel 4R and Tel 10R between experimental and computational studies may indicate that specific factors in addition to the modeled nuclear architecture may be involved. Furthermore, we found that when cell nuclei is enlarged to *D* = 4 and to *D* = 16 *μ*m, the two linear regimes between the median telomere distances disappear and becomes a single regime(Fig 2B).

The origin of the change in the slope has been attributed to the accessible area for telomeres. It was suggested in both experimental [39] and computational studies [26] that the accessible areas on the nuclear envelope for telomere attachment are limited by the arm lengths of short chromosomes, as they are anchored at SPB by the centromeres. Therefore, the median distance between telomeres increases as the chromosomal arm length increases, since it increases the accessible areas for telomere attachment. Once telomeres on chromosomes with arm length long enough to reach large areas on the NE, further increase in the chromosomal arm length does not significantly alter the spatial distances between telomeres [26, 39]. Overall, our results shows that for telomeres randomly located on the nuclear envelope, the size of their accessible areas is determined by both the chromosomal arm lengths and the nuclear diameter. It is the combination of these two geometrical factors that lead to the observed two regimes of telomere-telomere distances.

### Attachment of centromeres to SPB is a major determinant of inter-chromosomal interactions

Here we studied the effects of landmark constraints on the organization of yeast genome, through analyses of additional ensembles in Table 1. The overall correlation between the interaction frequencies from each ensemble and from experimental measurements is strong (*R* > 0.75, Table 1), suggesting again nuclear confinement and excluded-volume effects that are common to all four ensembles are the dominant factors in determining the overall interaction patterns of the budding yeast genome.

Inter-chromosomal interactions in most of ensembles are also highly correlated with experimentally captured inter-chromosomal interactions, except the ensembles in which centromere tethering is turned off. When the constraint of centromere tethering is removed, the correlation deteriorate from 0.75 to 0.30. These findings suggest that models with the constraint of centromere tethering to the SPB imposed in addition to the volume confinement can capture inter-chromosomal interactions observed in genome-wide 3C experiments. Indeed, we see an increase in the inter-chromosomal correlation with a negligible compromise in the intra-chromosomal correlation when we imposed only the centromere tethering as the constraint (Table 1). This also suggests a nonlinear correlation relationship between the number of constraints and the agreement with the experimental observation.

Specifically, when one or more constraints are removed while nuclear confinement and centromere tethering are maintained (Table 1, column 2–4 and column 6), the correlation between experimental and model data of inter-chromosomal interactions fluctuate somewhat, but all have high values (0.75–0.86). When the centromere constraint is removed, the correlation *R* deteriorates significantly to 0.30. Upon additional removal of nucleolus and telomere constraints, *R* further deteriorates to 0.25 (row 2, col 7).

For intra-chromosomal interactions (row 3), models with different constraints removed all show overall similar correlation (*R* = 0.87 – 0.95, col 2–6), and *R* = 0.89 when only the confinement and self-avoiding conditions are imposed (col 7). These slight fluctuations may be due to different sampling efficiencies, as it is easier to satisfy the constraints when the number of constraints decrease. Our findings show that specific landmark constraint affects the organization of budding yeast genome differently. The nucleolus constraint has effects only on the configurations of chromosome 12 (Fig D in S1 Text). We further examined the importance of centromere tethering on the pairwise distances between telomeres. When the centromeres are not attached to the SPB, the linear relationship between pairwise telomere distances and chromosomal arm lengths that was observed in fluorescence imaging experiments disappears (Fig C in S1 Text).

Overall, these results showed that centromere attachment to the SPB largely determines the chromosome-chromosome interactions, hence the chromosomal positioning in the nucleus. The folding landscape of individual chromosomes, on the other hand, is largely determined by the nuclear confinement and volume exclusion. Furthermore, our results show that not all constraints contribute equally to the overall organization of the budding yeast genome. Indeed, the removal of nucleolus constraint alone has minor influence on the correlation between experimentally measured and computed interactions. In contrast, our results showed that spatial confinement and centromere attachment play key roles in the genome organization of budding yeast.

### Spatial location of eight important genes are determined by their genomic distances to the centromeres

**Fig 3:**
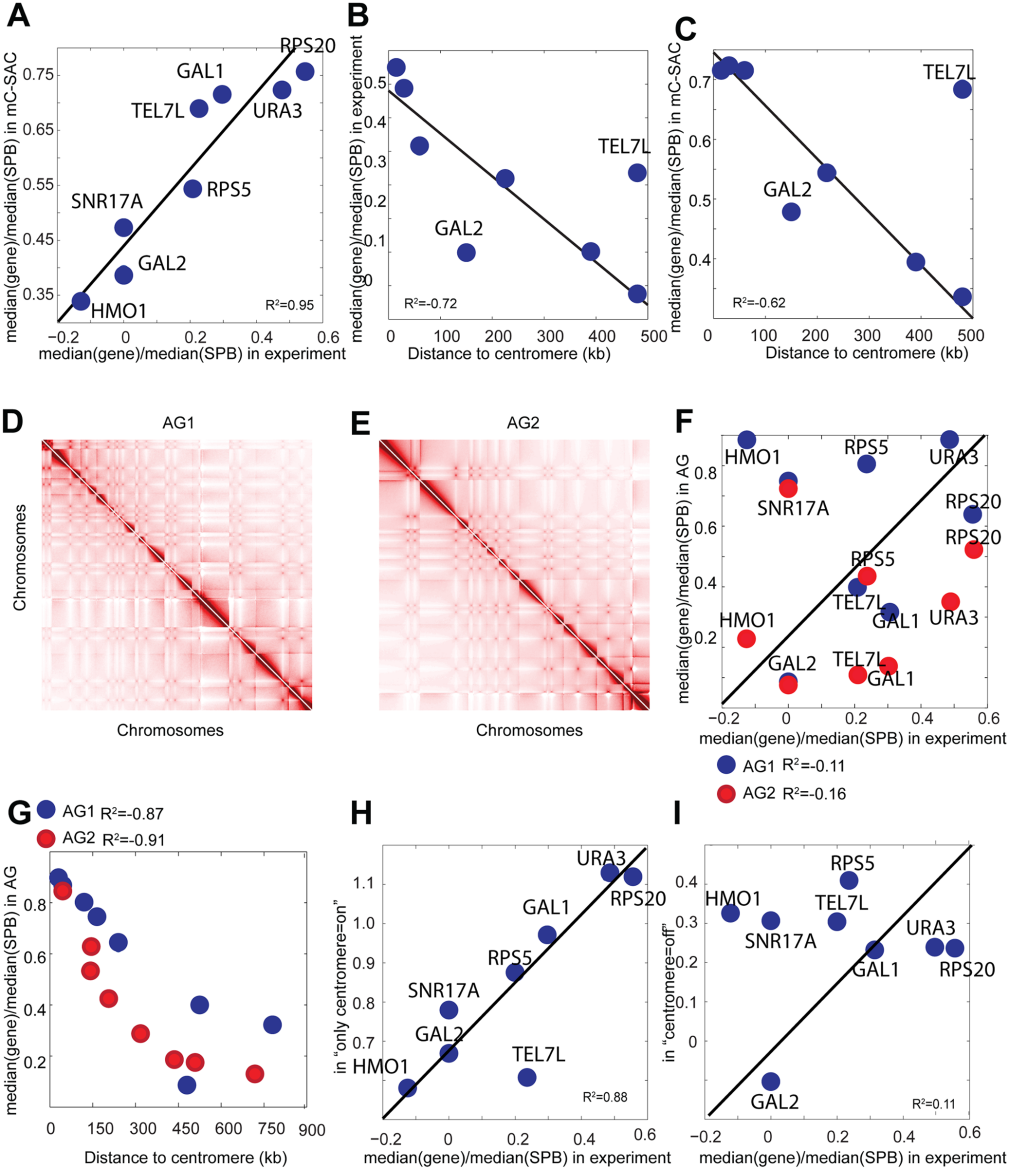
Relationship between genomic and spatial positions of eight genes. **(A)** The correlation between the relative positions of these genes measured by electron microscopy [6] (*x*-axis) and by fully-constrained ensemble (*y*-axis). **(B)** The relationship between the experimentally measured relative spatial positions of the important genes and their distance to the corresponding centromeres. The two locations of genes that correlate poorly are on Chr12 and telomere, which are subject to nucleolus and telomere attachment constraints. **(C)** The same relationship can be seen from computationally generated fully-constrained ensemble. **(D)** Heat map of interaction frequencies of Artificial Genome 1 (AG1) with 16 total chromosomes. **(E)** Heat map of interaction frequencies Artificial Genome 2 (AG2) with 12 total chromosomes. **(F)** The correlation between the relative position of the genes measured experimentally and measured from AG1 (blue) and AG2(red) ensembles. **(G)** The relationship between the relative positions of the genes measured from AG1 (blue) and AG2 (red) ensembles and their distances to the corresponding centromeres. The distances of these genes to their corresponding centromeres in artificial nuclei are different from each other and are all different from their corresponding distances in real yeast nuclei, as we assign random genomic coordinates to the centromeres in the artificial nuclei. **(H)** The correlation between the relative positions of the genes measured by electron microscopy [6] and by “with only centromere” ensemble. **(I)** The same correlation between the positions measured by electron microscopy [6] and in the “without centromere” ensemble.

The spatial locations of genes affect their transcriptional status [1]. The relative positions of seven important genes of the budding yeast and the left telomere of Chr 7 with respect to the SPB were measured in a fluorescence imaging study [6]. These genes include HMO1 on Chr 4, GAL2 on Chr 12, SNR17A on Chr 15, RPS5 on Chr10, GAL1 on Chr2, URA3 on Chr5, and RPS20 on Chr 8. Previous computational models showed an agreement between relative positions of modeled genomes and experimentally observed locations [26, 27]. We compared the positions of these genes measured from our fully-constrained ensemble with experimentally observed relative positions and found an agreement (*R*^2^ = 0.95, Fig 3A). Specifically, the relative positions of these genes are found to be inversely correlated with their genomic distances to corresponding centromeres, similar to a previous study [6] (Fig 3B and 3C). In the original imaging study, a gene located at (*a*_gene_, *b*_gene_, *c*_gene_) in the three–dimensional space is projected to two principal axes with coordinates of (*ρ*_gene_, *z*_gene_), where *ρ*_gene_ corresponds to the projection of (*b*_gene_, *c*_gene_), and *z*_gene_ corresponds to the cartesian location *a*_gene_ (Fig A in S1 Text). The relative position of a gene is calculated as the ratio of *a*_gene_/*a*_SPB_. Since the centromeres are located in the SPB, which is near the nuclear envelope (towards (*a*, *b*, *c*) = (−0.7, 0, 0) in Fig 1) and furthest away from the origin, a gene with genomic location away from the centromere would have its projected *z*-coordinate closer to that of the origin (*a*, *b*, *c*) = (0, 0, 0). For example, a gene with *a*_gene_ = −0.1 will have a ratio of −0.1/ − 0.7, which is smaller than the ratio of a gene that is located on SPB, as its ratio would be −0.7/ − 0.7. That is, the relative position of a gene to the SPB decreases as it becomes closer to the origin and its genomic distance to the centromere increases. We hypothesize that the relative positions are determined by the genomic distances of these genes to centromeres. To test this hypothesis, we generated two artificial genomes that have the same overall genome size and architecture as the budding yeast nucleus. Artificial Genome 1 (AG1) has the same number and lengths of chromosomes as the budding yeast genome, but with randomized locations of the centromeres. Artificial Genome 2 (AG2) has only 12 chromosomes, with the locations of centromeres also randomized. We found the same cross-like pattern in the interaction frequency heat map as the budding yeast genome for AG1 and AG2 (Fig 3D and 3E), suggesting that the number and the length of the chromosomes have little effects on the overall pattern of yeast genome organization.

However, when the genomic locations of the eight genes were mapped to the artificial genomes, their relative positions deviate significantly from the experimentally measured positions (*R*^2^ = 0.16 and *R*^2^ = 0.11 for AG1 and AG2, respectively, Fig 3F). Surprisingly, the inverse relationship between the genomic distance to the corresponding centromere and the relative positions of these genes observed in wild type yeast is well preserved (*R*^2^ = −0.87 and *R*^2^ = −0.91 for both artificial genomes, respectively, Fig 3G).

We further compared experimentally measured relative positions of these genes with their positions obtained from the ensembles of “with only centromere” and “without centromere” to examine the roles of centromere tethering on genome organization. The ensemble of “with only centromere” captured the relative spatial positions of these genes quite well (*R*^2^ = 0.88, Fig 3H), whereas the relative positions in the ensemble of “without centromere” do not correlate well with experimental measurements (*R*^2^ = 0.11, Fig 3I).

Overall, these results strongly suggest that centromere tethering is a key determinant of the folding of yeast genome and the positions of several important genomic elements are largely determined by their genomic distances to their corresponding centromeres.

### Chromosomal fragile sites are clustered in three-dimensional space

In eukaryotes, chromosomes can break at specific locations when DNA replication is perturbed [41]. These specific locations are called fragile sites. A recent genome-wide study of mapping of fragile sites showed that they are associated with sequence and structural motifs that pause or stall the DNA replication forks [41]. Fragile sites were also found to be associated with the origin of replication [42].

**Fig 4:**
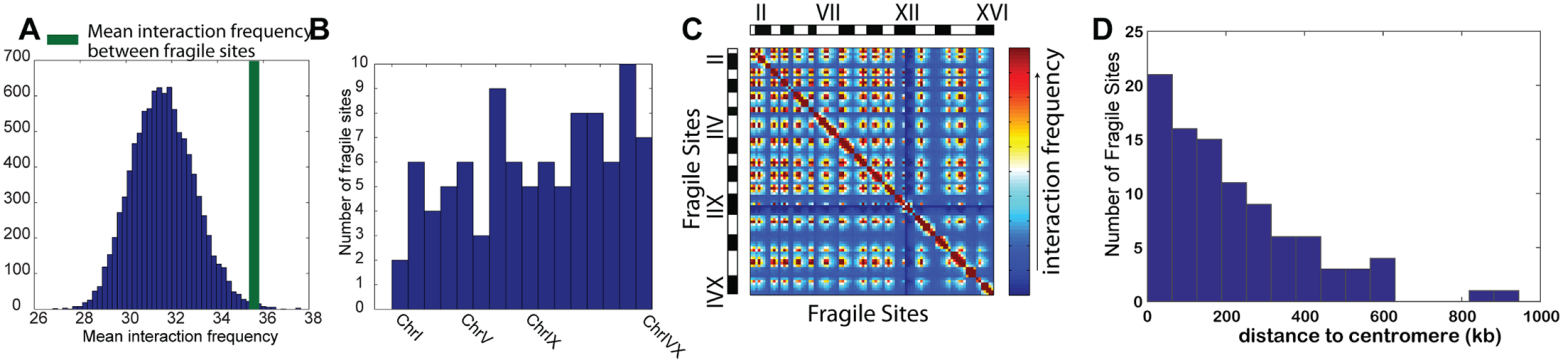
Interactions among fragile sites and their distribution in the budding yeast genome. **(A)** Mean interaction frequency between fragile sites (shown as thick green line) and the histogram of mean interaction frequencies between 10,000 sets of 95 random sites. **(B)** The distribution of fragile sites in the 16 chromosomes. **(C)** Heat map of interaction frequencies between fragile sites as computed from the fully-constrained ensemble. The length of each chromosome is proportional to the number of fragile sites it contains. All high frequency interactions (red) are predicted to occur between different chromosomes, except those on the diagonal. **(D)** The distribution of fragile sites by their genomic distances to the corresponding centromeres.

We mapped all 201 experimentally identified fragile sites to beads in our polymer model of yeast genome and calculated the mean interaction frequencies among them. Only non-local interactions between fragile sites that are more than 45 kb apart are considered, and proximity effects are eliminated in our consideration. Overall, the mean interaction frequency between the 95 mapped beads containing fragile sites is 35.9. The random probability of observing similar or higher frequency is *p* < 0.001 (Fig 4A), as estimated by bootstrapping 10,000 sets of 95 random beads that are at least 45 kb apart, and most of these interactions are found to be between different chromosomes. These results showed that fragile sites have a high propensity of clustering spatially together in the nucleus (Fig 4B and 4C), indicating that the underlying mechanism of double-stranded DNA breaks coming together in 3D space to create a repair foci [43] may be facilitated by the centromere tethering and the confinement of the cell nucleus. This is not surprising as majority of the fragile sites are located within 200 kb of the centromeres (Fig 4D) and is likely a result of centromere co-localization on the SPB.

### Predicting novel long-range chromatin interactions of budding yeast genome

While genome-wide 3C technique has identified many long-range pairwise chromatin interactions in budding yeast [16], these interactions are incomplete due to the distribution of restriction enzyme sites and lack of full mappability of the fragments. Our fully-constrained ensemble can be used to predict novel interactions that are not captured by genome-wide 3C experiments. In addition, it is also important to identify biologically specific interactions captured in genome-wide 3C studies but are unaccounted for by polymer effects under landmark constraints and nuclear confinement.

#### Predicted genomic interactions involving RNAPIII and TFIIS

**Fig 5:**
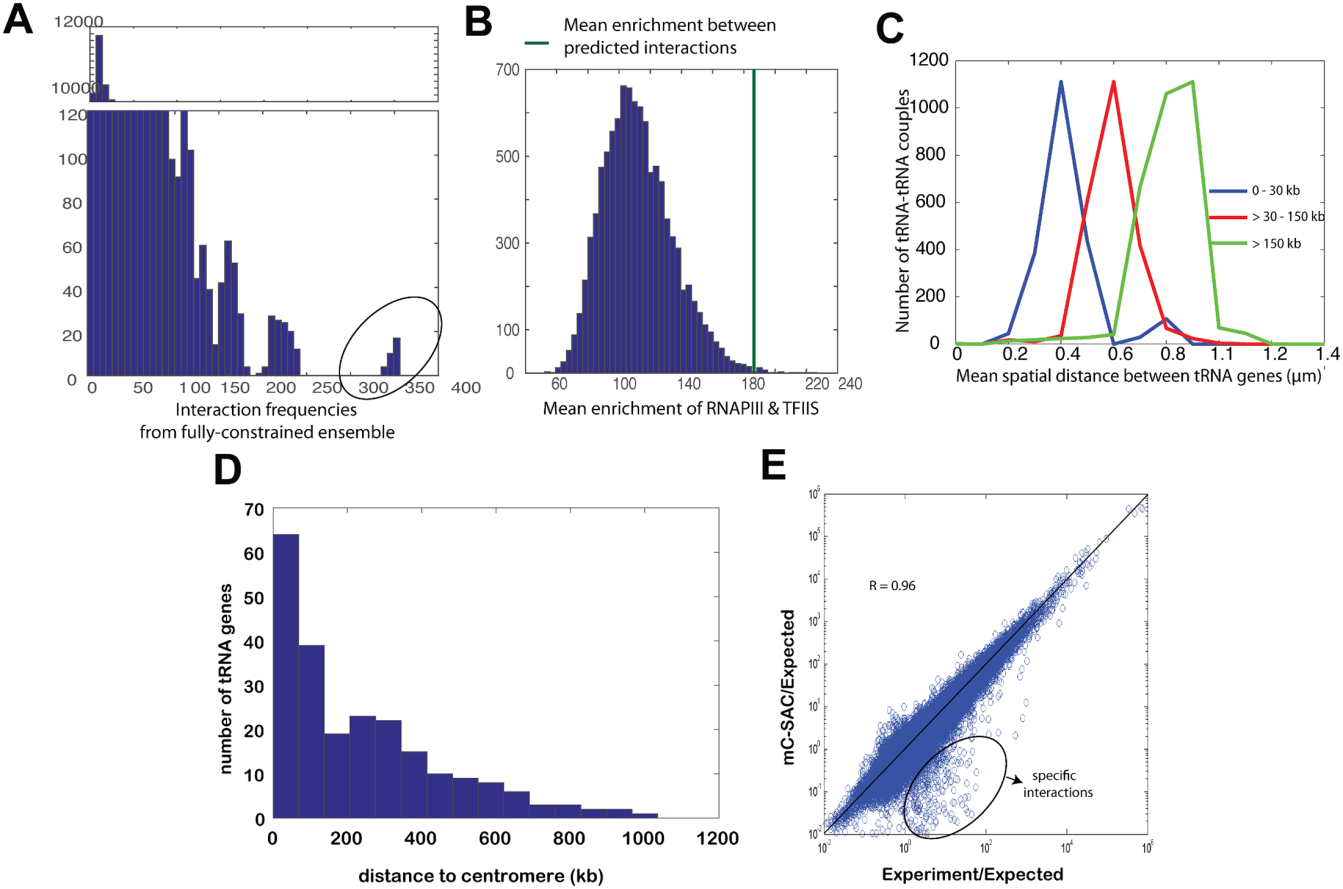
tRNA gene interactions and differentiating biologically specific interactions from non-specific interactions arising from polymer effects. **(A)** Distribution of frequencies of interactions enriched in the fully-constrained ensemble, but absent in the genome-wide 3C data. The 14 novel interactions with significantly more interaction frequencies are encircled. The *x*-axis values are the interaction frequencies and the *y*-axis values are the number of interactions that these frequencies are observed. **(B)** Histogram of enrichment factor of RNAPIII and TFIIS binding. Mean enrichment of predicted interactions are shown as the solid green line, along with the histogram of enrichment of 10,000 random sets of 14 interactions. **(C)** Distribution of mean-spatial distances between tRNA genes grouped according to their genomic distances to centromeres. **(D)** The distribution of tRNA genes by their genomic distances to the corresponding centromeres. **(E)** Interaction propensities of genome-wide 3C data (*x*-axis) and the fully-constrained ensemble (*y*-axis) calculated using a random ensemble as the null model. Interactions enriched in the genome-wide 3C data over the fully-constrained ensemble are enclosed in the black circle.

There are 14 interactions occurring between 10 loci that appear in more than 15% of the chains in the fully-constrained ensemble but are absent in the genome-wide 3C data (Fig 5A). We examined the available ChIP-chip study of RNAPIII and TFIIS binding ([44], see S1 Text) and found that there is an enrichment factor of 182.10 on average in binding of these factors to the 10 loci (see S1 Text). This is higher than the expected enrichment of 112.25 at a significance level *p* < 10^−2^ (Fig 5B), which is estimated from 10,000 sets of 14 random interactions of loci pairs. In addition, all 14 interactions are between centromeres and contain at least one tRNA gene (SI Table 1). Only 3 out of 14 interactions have enrichment of RNAPIII and TFIIS lower than the expected enrichment of random interactions (112.25). These findings are consistent with the observation of the centromeric localization of tRNA genes, which are transcribed by RNAPIII [45], as well as the association of elongation factor TFIIS with RNAPII that are important for tRNA gene expression [44]. Our results suggest that a subset of computationally predicted interactions may have originally arisen from confinement and landmark constraints, but were subsequently stabilized through evolution with binding of RNAPIII and binding of TFIIS. The abundance of tRNA genes involved points to likely biological roles of these genomic interactions.

#### Origin of tRNA-tRNA gene interactions

Genome-wide 3C experiments and polymer models strongly suggest that tRNA genes cluster together in 3D space [16, 26, 27]. However, the origin of this spatial clustering is unclear, as clustering could arise from the landmark constraints, or alternatively, from biological factors such as cohesin [46] and/or condensin [47]. After sorting all possible tRNA gene interactions according to their average separation distance from the corresponding centromeres, we find that mean spatial distances between tRNA genes are smaller when their average genomic distances from the corresponding centromeres are within 30 kb (Fig 5C). While specific association of condensin with tRNA gene complexes is suggested to mediate tRNA gene clustering in yeast nucleus [47], our results indicate that, to a large extent, the clustering of tRNA genes is likely a consequence of the spatial clustering of centromeres to the SPB. This is also supported by the distribution of the genomic distances of the tRNA genes to their corresponding centromeres (Fig 5D). Majority of tRNA genes are located within 200 kb of centromeres, hence their interactions with each other likely originate from the centromere co-localization in the SPB.

#### Biologically specific interactions beyond polymer effects

We further identify chromatin interactions measured by genome-wide chromosome conformation capture, but are unaccounted for by polymer effects and are likely biologically significant. We computed propensities of interactions in the fully-constrained ensemble and in the genome-wide 3C experimental measurements using the random ensemble under the constraint of confinement only as the null model. There are 19 experimentally captured interactions with a propensity ≥3.5 in genome-wide 3C data but <1 in the fully-constrained ensemble (Fig 5E, see also S1 Text). Among the 19 interaction pairs, 4 are between tRNA genes. To further confirm that these interactions are not due to polymer effects, we calculated the correlation of the frequencies of these 19 interactions between fully-constrained ensemble and genome-wide 3C data, which exhibit a small *R* value of 0.11. Furthermore, there are 70 important genes considered to be landmark genes in the budding yeast genome according to literature [48] (for a list, see SI Table 2). We found that 8 of the identified 19 specific interactions are between these landmark genes (see SI Table 3). Among these 8 pairs, the genetic interaction between genes CYS3 and ADE4 has already been recently reported [49], although the genetic relationship of the rest of the interacting landmark genes require further experimental investigations.

## Discussion

Eukaryotic genomes reside within the confined space of cell nucleus, and its organization is also directed by interactions with substructures called nuclear landmarks. Previous studies [26, 27] have already shown that random configurations of tethered chromosomes can reproduce measured interaction patterns [16] in the budding yeast genome, although the reported correlation between modeled and measured inter-chromosomal interactions is at the modest resolution of 32 kb, which is not strong. The direct effects of individual nuclear landmarks on genome folding, as well as the origin of inter-chromosomal interactions are unknown. A major technical challenge is the extreme difficulty in obtaining an adequate sampling of multiple chromatin chains subject to both landmark constraints and the confinement of the cell nucleus. The mC-SAC model developed in this study is based on a novel sampling technique [32, 33] to achieve this. It enables the generation of large ensembles of model genomes with different combinations of landmark constraints under nuclear confinement.

Our results showed that nuclear confinement and excluded-volume effects alone largely determine intra-chromosomal interaction patterns of individual yeast chromosomes, without the requirement of centromere tethering to the SPB and telomere attachment to the NE. This is in agreement with the results from polymer-diffusion studies [50]. Our results also highlight the importance of nuclear size on the patterns of interactions of genomic elements, as the experimentally captured interaction patterns disappeared, when the nuclear size is enlarged. Our results further demonstrated that centromere tethering to the SPB, along with the nuclear confinement and excluded-volume effect, are sufficient to capture the patterns of inter-chromosomal interactions. Furthermore, measured inter-chromosomal interactions are dominated by interactions between pericentromeric regions, hence a cross-like pattern originating from centromeres is observed. Our results also showed that, when only the landmark constraint of centromere tethering to the SPB is introduced, observed patterns of inter-chromosomal interactions are reproduced. Our results suggest that gene-regulatory systems involving long-range chromatin interactions might have been inherited from the telophase of budding yeast. Furthermore, the key difference in the regulatory machinery between the telophase and the interphase cells might be the silencing of telomeric genes through attachment to the NE. Such attachment, however, has no significant effects on the overall genome organization of budding yeast (Fig D in S1 Text).

Previous studies showed the presence of co-localization and clustering of important genomic elements such as early replicating sites or tRNA genes [16, 26]. However, the origin of such clustering remained unclear. Our results demonstrated that this clustering is largely due to the attachment of centromeres to the SPB. Except genes on Chr 12 and telomeres, positions of genomic elements on the chromosomes relative to the SPB are strongly correlated with their genomic distances to their corresponding centromeres. We also showed that the relative positions of genes can be reproduced, when the location of centromeres are randomized, and even when the total number of chromosomes artificially altered, as long as their genomic distances to the corresponding centromeres are given. This finding may be useful for predicting spatial positions of important genes from their genomic locations. For example, the spatial distances between tRNA genes decrease as their genomic distances to the centromeres decrease (Fig 5C). Our results are consistent with the suggestion that genomic locations of important elements in budding yeast were selected by evolutionary pressure [26].

Our model of budding yeast can be used to infer the biological details of the organization of yeast genome. The fully constrained ensemble can not only reproduce the pattern of spatial interactions from genome-wide 3C studies, but can also provide additional details by filling in the gaps in the sparse interaction matrices. Interactions arising from landmark constraints but absent in the genome-wide 3C data are enriched with transcription factors TFIIS as well as RNAPIII. These are located in pericentromeric regions of chromosomes, and contain significant amount of tRNA genes. In addition, we found that chromosomal fragile sites are clustered together in three–dimensional space, most likely as a result of their location at pericentromeric sites and a consequence of centromere clustering at the SPB. The proximate clustering of fragile sites suggest a machinery for DNA double break repair to repair multiple break sites, even those located on different chromosomes. It further suggests that these sites might experience less selective pressure to maintain resistance to perturbations. As SPB functionally corresponds to centrosome in mammalian cell nuclei, where the centromeres are attached during metaphase, our results may suggest that fragile sites of human genome could form spatial clusters and also be in genomic proximity to the centromeres. It is further possible that translocations due to the errors during mitosis in the human genome might be cancer promoting may also be related to centromere clustering.

Because of the dominant effects of landmark constraints and confinement on the folding patterns of the budding yeast genome, it is challenging to uncover the specific spatial interactions that are due to biological factors. One approach to identify such interactions is to generate ensembles of model genomes that are subject to landmark constraints. Taking this ensemble as a null model, one could in principle remove polymer effects from the interactions captured in genome-wide chromosome conformation capture study. However, current polymer models are inadequate for such a task, as they cannot reproduce the inter-chromosomal interaction patterns, and hence will introduce many false positives [26, 27]. Previous studies also suggested that volume exclusion models capture only expected interactions when such expected interactions were removed, as there were no significant correlations between model genomes and experimental measurements [31]. Our results suggest that such correlations can be improved significantly with better sampling techniques. To further understand whether the budding yeast genome organization is dictated by landmark constraints, we removed the interactions arising from excluded-volume effects, chain connectivity and nuclear confinement from both experimental measurements and our fully-constrained computed ensemble, and compared the remaining interaction frequencies. Our results suggest that overall experimentally measured interactions are in agreement with the remaining interactions of the fully-constrained ensemble of modeled genomes. Furthermore, there exists a set of interactions that occur at high frequency in the genome-wide 3C data but are almost absent in the fully-constrained ensemble. These interactions involve several important genes. Overall, we were able to extract interactions of potential biological interest from the interaction frequencies of genome-wide 3C data, a challenging task due to the dominance of polymer effects in experimental measurements. These interactions are found to be between some of the tRNA genes as well as landmark genes.

With improved mC-SAC sampling technique, our computed 3D ensembles of budding yeast genome recaptures the observed intra- and inter-chromosomal interactions at the finer resolution of 15 kb, a resolution higher than those of previous studies [26, 27]. Our study also reveals a number of novel findings that were not previously seen [26, 27]. First, our results showed that spatial confinement and excluded volume effects alone can account for measured intra-chromosomal interactions. Second, attachment of centromeres to SPB is a major determinant of inter-chromosomal interactions, which was not accounted for in previous studies (R = 0.75 in this study *vs.* R = 0.54 in [26]). Third, spatial locations of eight important genes can be determined by their genomic distances to the centromeres, as genomic distance of loci to centromeres and their spatial locations are now shown to be highly correlated. Fourth, chromosomal fragile sites, defined as double-stranded DNA breaks upon DNA perturbation, are found to be cluster in three-dimensional space. Fifth, we predicted novel long-range chromatin interactions not present in genome-wide 3C study that are mediated by RNAPIII and TFIIS, all involving tRNA genes. Sixth, our results confirm recent finding of tRNA gene clustering largely from centromere attachment to SPB. Finally, we succeeded in removing expected interactions from experimental measurements and identified important biologically specific genome-wide 3C interactions beyond any polymer effects. While these are important findings, our model is still limited, as it does not contain sufficiently detailed spatial information, because of the coarse-grained nature of both the mC-SAC model and the available genome-wide 3C data on budding yeast genome. Inferring the structural details of gene regulation for just a few kilo-bases requires chromatin models of much finer resolution. This finer resolution awaits advances in theory, model, and experimental measurements.

## Supporting information

S1 Text Additional information on mC-SAC algorithm and further details of analysis of model genomes. (PDF)
