# Correction: Spatial organization of the budding yeast genome in the cell nucleus and identification of specific chromatin interactions from multi-chromosome constrained chromatin model

**DOI:** 10.1371/journal.pcbi.1005778

**Published:** 2017-10-02

**Authors:** The PLOS Computational Biology Staff

The funding statement for this article should read as follows:

“This work is supported by National Institute of Health (https://www.nih.gov) GM079804, GM126558, AI126308-01A1 and the Chicago Biomedical Consortium (http://www.chicagobiomedicalconsortium.org/) with support from the Searle Funds at The Chicago Community Trust. We also thank the Research Open Access Publishing (ROAAP) Fund of the University of Illinois at Chicago for financial support. The funders had no role in study design, data collection and analysis, decision to publish, or preparation of the manuscript.”
