# Supplementary material for: Spatial organization of the budding yeast genome in the cell nucleus and identification of specific chromatin interactions from multi-chromosome constrained chromatin model
Source: PLoS Comput Biol. 2017 Jul 13;13(7):e1005658. doi: 10.1371/journal.pcbi.1005658 (PMC5531658; doi:10.1371/journal.pcbi.1005658)
Supplement: S1 Text — (PDF) [file pcbi.1005658.s001.pdf]

Supplementary Information

Gamze Gürsoy[1], Yun Xu[1] and Jie Liang*[1]

[1]The Richard and Loan Hill Department of Bioengineering, University of Illinois at Chicago, Chicago, IL 60607, USA

July 1, 2017

*jliang@uic.edu, (312) 355 1789; Corresponding Author

Supplementary Methods

Normalization and calculation of propensity

We normalized the interaction frequencies of experiments and the model ensembles following the previous work [1, 2, 3]. Let f_{ij} be the interaction frequencies between the genomic elements i and j. We obtained the normalized interaction frequency as

$$f_{ij}^{n} = f_{ij} \times \frac{\sum_{k=1}^{N} \sum_{l=k+1}^{N} f_{kl}}{\sum_{k=1}^{N} f_{ik} \sum_{k=1}^{N} f_{kj}},$$

where N is the total number of the genomic elements. All the calculations in this paper are employed after normalization of experimental and model ensembles.

The propensity of an interaction is the observed/expected for the experiment and the modeled ensembles. First, we calculated the probability of an interaction in the experimental interaction matrix, interaction matrix of modeled ensemble and random model as following,

$$q^{\mathrm{exp}}(ij) = \frac{f^{exp}(ij)}{\sum_{i=1}^{N} \sum_{j=1}^{N} f^{exp}(ij)}$$

$$q^{\mathrm{model}}(ij) = \frac{\sum_k w_k I(i,j)}{\sum_{i=1}^{N} \sum_{j=1}^{N} \sum_k w_k I(i,j)},$$

$$q^{\mathrm{random}}(ij) = \frac{\sum_k w_k I(i,j)}{\sum_{i=1}^{N} \sum_{j=1}^{N} \sum_k w_k I(i,j)},$$

where N is the total number of genomic elements, w_k is the weight of the $k^t h$ chain in the ensemble and $I(i,j)$ is an indicator function, which equals to 1 when elements i and j interacts, equals to 0 otherwise. q^{model} is calculated using the model genomes in the constrained models, whereas q^{random} is calculated using the model genomes in the random ensemble. We calculated the propensity of each interaction as,

$$propen^{\text{exp}}(ij) = \frac{q^{\text{exp}}(ij)}{q^{\text{random}}(ij)}$$

$$propen^{\text{model}}(ij) = \frac{q^{\text{model}}(ij)}{q^{\text{random}}(ij)}$$

Calculation of p-value for the correlation between experimental matrix and model ensemble matrix

We shuffled the each row of the experimental interaction matrix for 1000 times and generated 1000 shuffled interaction matrices. We calculated the mean row-based Pearson correlation coefficient between each shuffled matrix and the modeled ensemble and calculated the probability of obtaining mean row-based Pearson correlation coefficient of 0.95 as the p-value (SI Fig. 2).

Mean combined occupancy enrichment

We mapped the genome-wide occupancy enrichment of RNAPIII and TFIIS on to beads. We used a geometrical mean approach for the coupled enrichment of pairs. Mean enrichment value for each pair is calculated as

$$en_{mean}(i) = \sqrt{\sqrt{(en_{RNAPIII}(i) * en_{RNAPIII}(j)} * \sqrt{en_{TFIIS}(i) * en_{TFIIS}(j)}}$$

Details of g-SIS algorithm

Target distribution

The target distribution $\pi(x)$ is the Boltzmann distribution of all chromosome chains that that are self-avoiding, with their centromeres attached to the SPB, the rDNA repeats placed in the nucleolus and telomeres attached to NE. The target distribution of a partial chain $\pi(x_t^k)$ follows Boltzmann

distribution as

$$\pi(x_t^k) = \exp(-E(x_t^k)/k_B T),$$

where $E(x_t^k)$ is an energy like term that is derived from the landmark constraints.

The target distribution $\pi(x_t^k)$ of a partial chain follows Boltzmann distribution as

$$\pi(x_t^k) = \exp(-E(x_t^k)/k_B T),$$

$$E(x_t^k) = H_1(x_t^k) + H_2(x_t^k)$$

(1) Potential from telomere closing constraints This potential is designed to obtain model genomes where the telemores are either attached to the NE when the full arm length is reached or can be attached to the NE at any point of chain growth,

Let $H_1(x_t^k)$ be the potential from telomere closing probability constraints. For each candidate node x_{t_m} that does not violate the self-avoiding property and inside the nuclear confinement, we calculate the energy-like term as

$$H_1(x_t^k) = |||| x_{t_m} || - R| - (N - t) \times L_p - d_{thres}|, \tag{1}$$

where L_p is the persistence length, N is the total number of nodes in a chromosomal arm, R is the nuclear radius and d_{thres} is the threshold distance which was taken as 50 nm.

(2) Potential from centromere tethering constraints. This potential is used only for the Chr12 chromosomal arms where the rDNA repeats are sampled from nucleolus and designed to obtain model genomes where the centromere are either in the SPB when the full arm length is reached or can be in the SPB at any point of chain growth,

Let $H_2(x_t^k)$ be the potential from centromere tethering constraints. For each candidate node x_{t_m}

that does not violate the self-avoiding property and inside the nuclear confinement, we calculate the energy-like term as

$$H_2(x_t^k) = ||\| x_{t_m} - x_{SPB} \| - R_{SPB}| - (N-t) \times L_p|, \quad (2)$$

where x_{SPB} is the center coordinates of SPB, L_p is the persistence length, N is the total number of nodes in a chromosomal arm, and R_{SPB} is the radius of SPB as we modeled as a sphere.

Trial distribution

Trial distribution is designed to introduce the necessary bias to generate partial chain x_t^k with the probability approximating to the target distribution $\pi(x_t^k)$. The trial distribution $g(x_t^k)$ of a partial chain x_t^k is

$$g(x_t^k) = \exp(\pi(x_t^k) - \max_{t=1,\ldots,1640} \pi(x_t^k))$$

.

Random model

A random ensemble of 150,000 model genomes with only excluded volume constraint and nuclear confinement are generated. To improve the sampling efficiency, we employ a dynamic resampling technique that has been described previously [4].

Statistical properties of model genomes

With m successfully generated model genomes, the physical properties of the ensembles of model genomes are calculated. If the configurations of the j-th successfully generated model genome as $x^{(j)} = (x_1^{(j)}, \cdots, x_n^{(j)})$, and its associated weight $w^{(j)}$. To calculate the mean value of a physical

Algorithm 1 Sequential Importance Sampling algorithm for mC-SAC model

Set $w_1^{(1)} = 1.0$
for $i = 1 \rightarrow n$ **do**
    Place all the centromere at fixed $x_i 1^{(1)}$
end for
Set a = 1
for $t = 2 \rightarrow n$ **do**
    while $a < N$ **do**
    // N: number of the chromosomal arms Choose a random chromosome arm i in the genome
        if $t \leq n_i$ **then**
    // n_i: length of chromosome arm n_i
            Find all of the valid sites $x_{it}^{(k,j)}$, $k = 1, \cdots, l_t^{(j)}$ for placing $x_{i,t}$ next to partial chain $\boldsymbol{x}_{i,(t-1)}^{(j)}$
            Generate $l_t^{(j)}$ number of t-length chromosome arm
            for $k = 1 \rightarrow l_t^{(j)}$ **do**
                Calculate target distribution $\pi(x_{i,t}^{(k,j)})$
                Calculate trial distribution $g(x_{i,t}^{(k,j)})$
            end for
            Select sample $\boldsymbol{x}_{i,t}^{(j)}$ with $\max_{k=1,\ldots,l_t^{(j)}} g(x_{i,t}^{(k,j)}))$
            Set weight $w_t^{(j)} = \widetilde{w}_t^{(j)} \cdot (g(x_{i,t}^{(j)}))/\pi(x_{i,t}^{(j)}))$
            Set $a = a + 1$
        else
            Set $a = a + 1$
        end if
    end while
end for

property $\bar{h}(x)$ such as the spatial distance between genomic elements, we have:

$$\bar{h}(x) = \mathbb{E}_{\pi(x)}[h(x)] = \frac{\sum_{j=1}^{m} h(x^{(j)}) \cdot w^{(j)}}{\sum_{j=1}^{m} w^{(j)}}.$$

Figure A: Schematic representation of the cell nucleus with the landmarks. (A) Yellow chromosome represents the Chr12 where the rDNA elements are highlighted as blue spheres and the centromere is highlighted as red sphere. Purple chromosome represents the rest of the chromosomes where centromere is highlighted as red sphere. The direction of chain growth is shown with the arrows. (B) Schematic representation of the chromosomes and the special case of Chr 12 where we used 3 chromosomal arms for chain growth process. (C) Schematic representation of projection of three-dimensional coordinates to two principal axis.

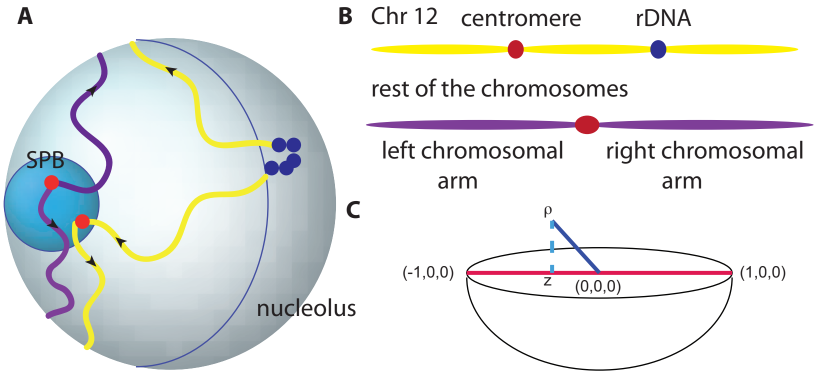

Figure B: Histogram of the mean row-based correlation coefficients between shuffled experimental data and the model ensemble.

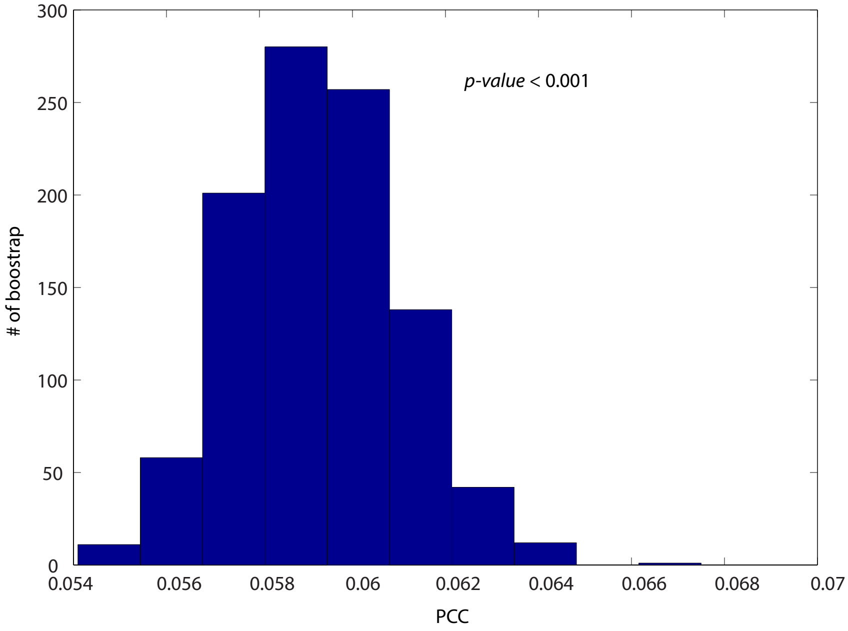

Figure C: The effect of centromere tethering on the median distances between telomeres. **(A)**Relationship between chromosome arm length and median telomere distances for the random model. No correlation between arm length and the median telomeric distances was observed. **(B)** Relationship between chromosome arm length and median telomere distances for the "centromere=off" ensemble. No correlation between arm length and the median telomeric distances was observed.

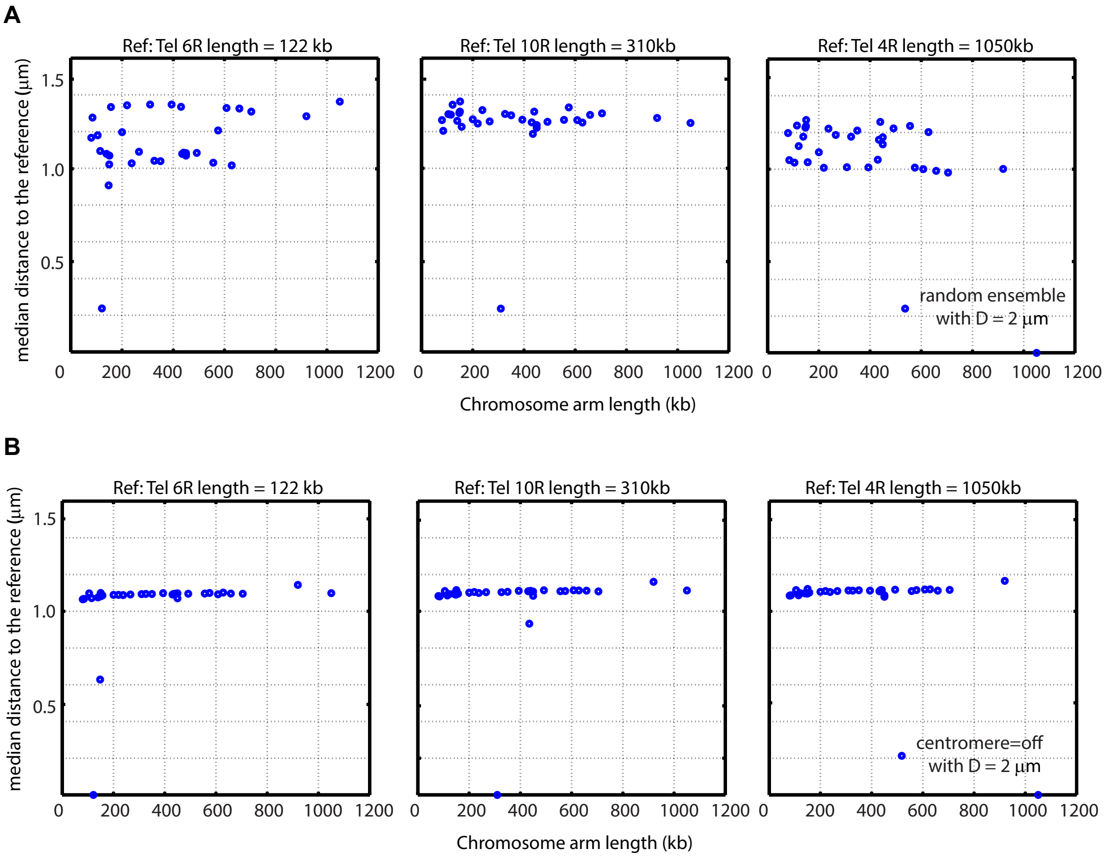

Figure D: Effects of different constraints on the interaction profiles of different genomic elements. (A) Interactions between the centromere of Chr II and the other genomic elements in the yeast genome. The interactions between the centromere of Chr II and the other centromeres are the same for the ensembles, in which centromere constraint is on. Despite the high-correlation coefficient between the experiment and the ensemble of without centromere, this ensemble fails to capture the centromere-centromere interactions. (B) Interactions between the telomere of Chr VII and the other genomic elements in the yeast genome. The interactions between the telomere of Chr VII and the other telomeres are the same for the ensembles, in which telomere constraint is on. However, since the frequency of telomere-telomere interaction is low, the ensembles, in which telomere constraint is off still have high correlation with Hi-C data. (C) Interactions between a random locus on Chr XII and the other genomic elements in the yeast genome. The intra-chromosomal interactions within Chr XII differs for all the ensembles.

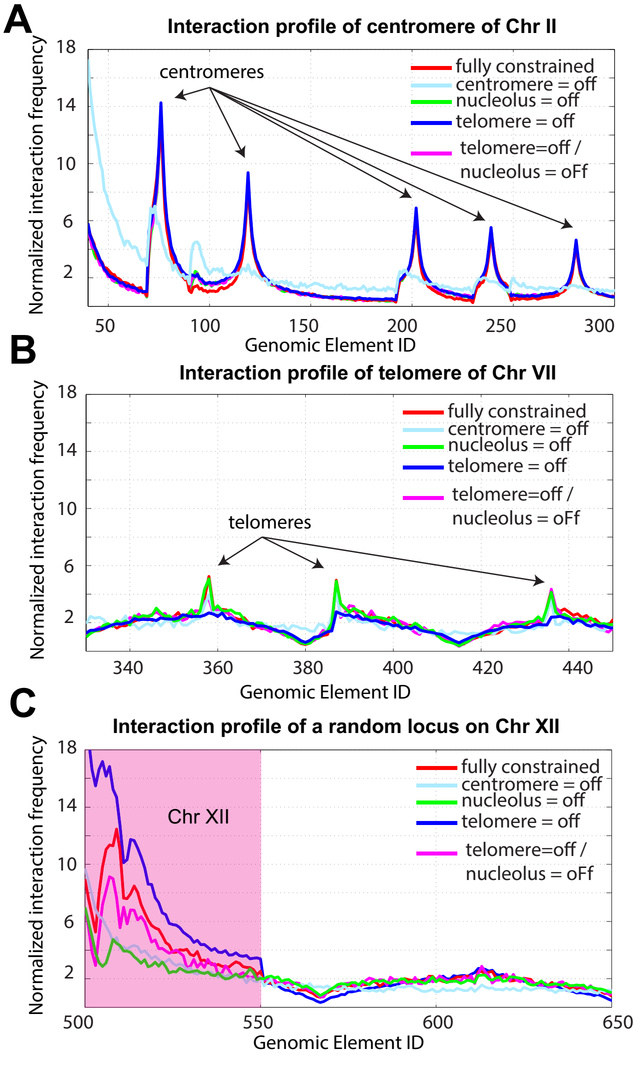

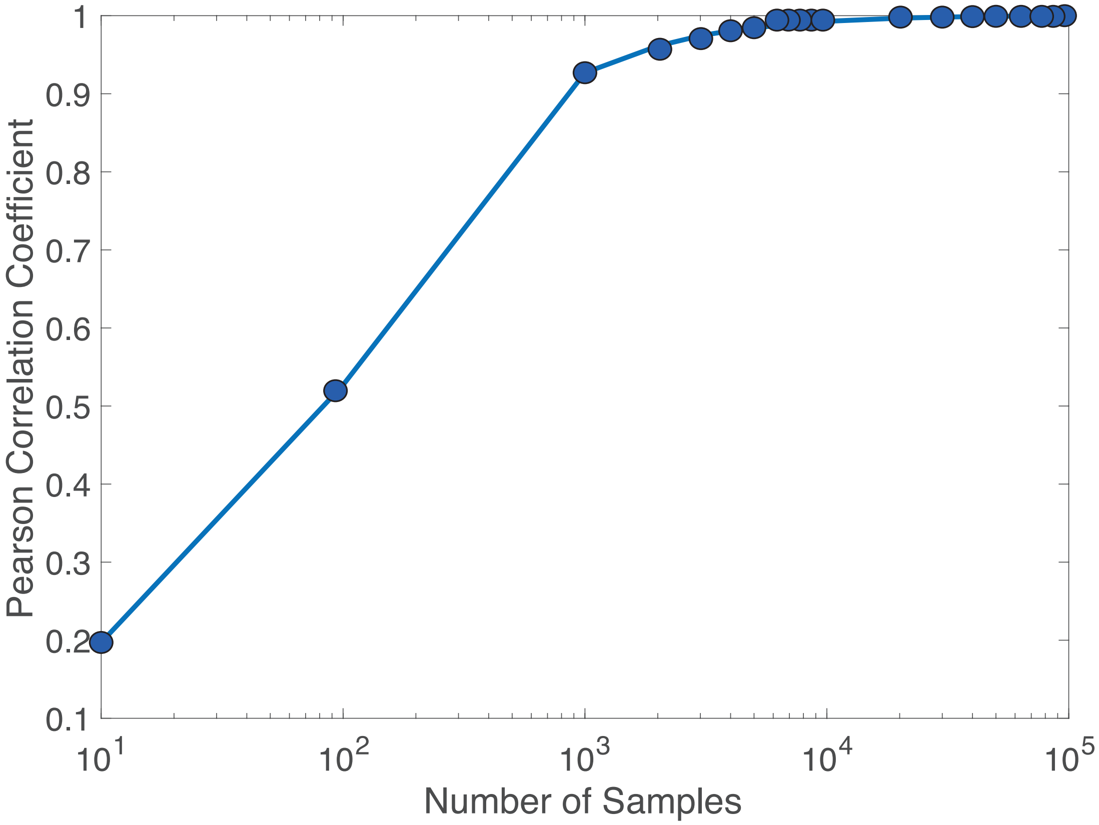

Figure E: **Conversion of ensemble by the sampled number of chains.** The Pearson Correlation between the interaction frequencies of full ensemble and partial ensembles with different number of models.

Table 1: Predicted 14 interactions between centromeres of chromosomes, whether they contain tRNA gene, and their combined enrichment value of RNAPIII and TFIIS

Chr	tRNA gene	Chr	tRNA gene	enrichment
II	yes	XIII	yes	56.98
II	yes	XV	yes	127.24
II	yes	XVI	no	61.53
IV	yes	XVI	no	163.63
V	yes	XVI	no	204.64
VII	no	XV	yes	183.53
X	yes	XIV	yes	372.52
X	yes	XV	yes	373.15
X	yes	XVI	no	180.46
XI	no	XV	yes	134.95
XIII	yes	XV	yes	146.34
XIII	yes	XVI	no	70.77
XIV	yes	XV	yes	326.23
XV	yes	XVI	no	158.03

Table 2: Landmark genes that are specified in the yeast database.

Chr1	FLO9, CLN3, MAK16, CYS3, ADE1, PHO1
Chr2	ILS1, MCM2, RAD16, SUP45, MET8
Chr3	HMLALPHA1, MATALPHA1, HMRA1
Chr4	CDC9, CDC2, SIR4, XRS2, TRP1
Chr5	CAN1, CUP5, FCY2, MET6, RAD3
Chr6	YPT1, SMC1, HIS2, HXK1
Chr7	ADH4, CUP2, TRP5, GCD2, PFK1
Chr8	SPOII, ARD1, CUP11, FUR1, ERG9
Chr9	SUC2, HIS5, BCY1, LYS1
Chr10	TPK1, ARG3, CYR1, CYC1, ECM17
Chr11	URA1, APE2, ELM1, VPS1, SIR1
Chr12	CDC25, LEU23
Chr13	HMG1, NDC1, MCM1, PFK2, ADE4
Chr14	DAL82, KEX2, RPC31, TOP2, LYS9
Chr15	HXT11, TOP1, DED1, PPO2, RAD17
Chr16	GAL4, TPK2, PEP4, ERG10, HTS1, RPC40

Table 3: Predicted interacting landmark genes. Each row contains a pair of interacting genes, identified from genome-wide 3C measurements using fully-constrained ensemble as null model.

gene	gene
CYS3	ADE4
TRP1	TOP2
SMC1	CYC1
SPOII	CYC1
FUR1	PEP4
ARG3	RAD17
MCM1	PEP4
PFK2	TOP1

References

[1] H. Tjong, K. Gong, L. Chen, and F. Alber. Physical tethering and volume exclusion determine higher-order genome organization in budding yeast. *Genome Research*, 22(7):1295–1305, July 2012.

[2] Erez Lieberman-Aiden, Nynke L. van Berkum, Louise Williams, Maxim Imakaev, Tobias Ragoczy, Agnes Telling, Ido Amit, Bryan R. Lajoie, Peter J. Sabo, Michael O. Dorschner, Richard Sandstrom, Bradley Bernstein, M. A. Bender, Mark Groudine, Andreas Gnirke, John Stamatoyannopoulos, Leonid A. Mirny, Eric S. Lander, and Job Dekker. Comprehensive mapping of long-range interactions reveals folding principles of the human genome. *Science (New York, N.Y.)*, 326(5950):289–293, October 2009.

[3] Zhijun Duan, Mirela Andronescu, Kevin Schutz, Sean McIlwain, Yoo Jung Kim, Choli Lee, Jay Shendure, Stanley Fields, C. Anthony Blau, and William S. Noble. A three-dimensional model of the yeast genome. *Nature*, 465(7296):363–367, May 2010.

[4] Gamze Gürsoy, Yun Xu, Amy L. Kenter, and Jie Liang. Spatial confinement is a major determinant of the folding landscape of human chromosomes. *Nucleic Acids Research*, 42(13):8223–8230, July 2014.
